# The von Hippel-Lindau Tumor Suppressor Gene Mutations Modulate Lipocalin-2 Expression in Ferroptotic-Inflammatory Pathways

**DOI:** 10.1155/2023/7736638

**Published:** 2023-01-21

**Authors:** Chan-Yen Kuo, Po-Chun Hsieh, Valeria Chiu, Chou-Chin Lan, Kuo-Cheng Lu

**Affiliations:** ^1^Department of Research, Taipei Tzu Chi Hospital, Buddhist Tzu Chi Medical Foundation, New Taipei City 231, Taiwan; ^2^Department of Nursing, Cardinal Tien College of Healthcare and Management, New Taipei City 231, Taiwan; ^3^Department of Medical Technology, Jenteh Junior College of Medicine, Nursing and Management, Miaoli, Taiwan; ^4^Department of Chinese Medicine, Taipei Tzu Chi Hospital, Buddhist Tzu Chi Medical Foundation, New Taipei City, Taiwan; ^5^Division of Physical Medicine and Rehabilitation, Taipei Tzu Chi Hospital, Buddhist Tzu Chi Medical Foundation, New Taipei City 231, Taiwan; ^6^School of Medicine, Tzu Chi University, Hualien 970, Taiwan; ^7^Division of Pulmonary Medicine, Taipei Tzu Chi Hospital, Buddhist Tzu Chi Medical Foundation, New Taipei City 231, Taiwan; ^8^Division of Nephrology, Taipei Tzu Chi Hospital, Buddhist Tzu Chi Medical Foundation, New Taipei City 231, Taiwan; ^9^Division of Nephrology, Department of Medicine, Fu-Jen Catholic University Hospital, School of Medicine, Fu-Jen Catholic University, New Taipei City 243, Taiwan

## Abstract

A previous study of an animal model with tumor suppressor gene von Hippel-Lindau (VHL) conditional knockdown suggested that tissue inflammation and fibrosis play important roles in the development of clear-cell renal cell carcinoma (ccRCC), which is consistent with the epidemiological evidence linking inflammatory kidney disease and renal cancer. Ferroptosis and inflammation have been linked in a recent study, but the exact mechanism remains unclear. This study is aimed at investigating the mechanism of lipocalin-2- (LCN-2-) mediated ferroptosis and inflammation in *vhl*-mutated HK-2 cells and mouse primary proximal tubule cells (mRTCs) and the polarization of macrophage RAW 264.7 cells. Based on the levels of lipid reactive oxygen species (ROS) and the expression of glutathione peroxidase 4 (GPX4) in HK-2 cells, we observed that a VHL mutation increased ROS production and depressed GPX4 expression, whereas LCN-2 knockdown reversed these effects. Accordingly, VHL appears to affect ferroptosis in an LCN-2-dependent manner. We also revealed that LCN-2 sensitizes HK-2 cells to inflammation and macrophage RAW 264.7 cells to M1-like polarization. This study provides novel insights into the potential therapeutic target and strategy for attenuating the progression of ccRCC by revealing the role of VHL in regulating chronic inflammation within the LCN-2–ferroptosis pathway.

## 1. Introduction

von Hippel-Lindau (VHL) mutations play a critical role in clear-cell renal cell carcinoma (ccRCC) development [1, 2]. A previous study reported the accumulation of large deposits of collagen fibers within clusters of distorted tubules in the kidneys of mice lacking VHL [3]. Moreover, we previously demonstrated that treatment with an inhibitor of serine/threonine protein kinase/endoribonuclease (IRE1*α*) alleviates inflammation and fibrosis in VHL-knockout mice [4]. Chronic inflammation has been suggested to mediate carcinogenesis in VHL mutation-associated ccRCC. The merging of tumor cells with the surrounding stromal and inflammatory cells creates inflammatory tumor microenvironments [5]. Although the mortality rate of patients with VHL disease has reduced due to the availability of genetic screening and early detection, the risk of metastatic ccRCC that can escape early detection remains, and there is no effective treatment for malignant ccRCC [6]. Therefore, the development of anticancer treatments will rely partly on understanding the molecular and cellular mechanisms that promote tumor growth.

Tumor biology and iron metabolism are closely related. Iron mediates the production of oxygen radicals, which can either cause ferroptosis or contribute to mutagenicity and malignancy [7]. Ferroptosis is a programmed or regulated cell death discovered by Dr. Brent Stockwell [8]. It is an iron- and radical oxygen species- (ROS-) dependent but a caspase-independent form of nonapoptotic cell death [8–10]. Of note, ferroptosis has a positive effect on inflammation by regulating immunogenicity [11]. The Food and Drug Administration has approved certain drugs that induce iron-mediated apoptosis, such as sorafenib, sulfasalazine, and artesunate, suggesting that ferroptosis might be a potential therapeutic strategy for cancer [9, 12]. In a xenograft model, tumor growth was attenuated after administering erastin, piperazine erastin, and RSL3 [9, 13, 14]. Accumulating evidence has demonstrated that glutathione peroxidase 4 (GPX4) is a key regulator of ferroptosis, an essential regulator of ferroptotic cancer cell death [13, 15, 16], and an inflammatory mediator [17, 18]. Depleting GPX4 was shown to enhance the expression of 12-lipoxygenase and cyclooxygenase 1 in a human carcinoma cell line, which causes inflammation [19]. Li et al. reported that GPX4 activation inhibits ferroptosis and inflammation by suppressing arachidonic acid oxidation and NF-*κ*B (nuclear factor-*κ*B) pathway activity, which reduces ROS production [20]. The exact mechanism remains unknown despite emerging links between ferroptosis and inflammation.

Some studies have demonstrated that lipocalin 2 (LCN-2) is an essential iron transport protein during physiological and inflammatory states [21]. In our previous study, we revealed the role of VHL in regulating inflammation in HK-2 cells via the LCN-2–ROS pathway [22]. Moreover, our findings suggested that LCN-2 triggers inflammatory responses in HK-2 cells and the chemotactic ability of macrophages [22]. Wang et al. reported that LCN-2 knockdown protects lipopolysaccharide-induced neonatal acute respiratory distress syndrome model by inhibiting ferroptosis-related oxidative stress and inflammation through MAPK/ERK signaling [23]. However, VHL's critical role in regulating ccRCC via the ferroptotic–inflammatory pathway is poorly understood. Therefore, this study sought to investigate the mechanism of LCN-2-mediated ferroptosis and inflammation in *vhl* mutation HK-2 cells. Furthermore, we speculated that LCN-2 is related to *vhl* mutation-sensitized RAW cell polarization in an LCN-2-dependent manner.

## 2. Materials and Methods

### 2.1. Reagents and Antibodies

Liproxstatin-1 (Lipro-1) was obtained from Sigma (St. Louis, MO, USA). Rhodamine B-((1,10-phenanthroline-5-yl-) aminocarbonyl) benzyl ester (RPA) was purchased from Squarix GmbH (Elbestr, Germany). Deferoxamine mesylate (DFO) salt was provided by Sigma (D9533). A 0.1 mM solution of DFO in phosphate-buffered saline (PBS, Sigma) was used as the solvent in this study. Controls were given the same volume of vehicle (dimethyl sulfoxide or PBS).

The rabbit polyclonal antibodies were used in the western blot analyses including Gpx4 (#A1933, 1 : 1000 dilution, ABclonal, MA, USA); LCN-2 (#A2092, 1 : 1000 dilution, ABclonal, MA, USA); VHL (#A0377, 1 : 500 dilution, ABclonal, MA, USA); *β*-actin (# 4967, 1 : 1000 dilution, Cell Signaling, MA, USA); pJNK (#4668, 1 : 1000 dilution, Cell Signaling, MA, USA); JNK (#9252, 1 : 1000 dilution, Cell Signaling, MA, USA); GAPDH (#AC001, 1 : 3000 dilution, ABclonal, MA, USA); iNOS (#13120, 1 : 1000, Cell Signaling, MA, USA); CD86 (#A19026, 1 : 750 dilution, ABclonal, MA, USA); IL-8 (#A2541, 1 : 500 dilution, ABclonal, MA, USA); arginase (#A1847, 1 : 500 dilution, ABclonal, MA, USA); ACSL4 (#A20414, 1 : 1000 dilution, ABclonal, MA, USA); HMGB1 (#A2553, 1 : 750 dilution, ABclonal, MA, USA); Lon (#A4293, 1 : 2000 dilution, ABclonal, MA, USA).

### 2.2. Cell Culture

Human renal proximal tubular epithelial cells (HK-2) and human embryonic kidney cells 293 (HEK293) were obtained from the BCRC (Bioresource Collection and Research Center; Taiwan) and cultured in Corning T75 flasks (Corning; NY, USA) in Dulbecco's Modified Eagle's Medium (DMEM)/Ham's F12 (Gibco; NY, USA) containing 10% heat-inactive fetal bovine serum, 2 mM glutamine, 25 mM glucose, 1 mM sodium pyruvate, 1 mM sodium pyruvate, and penicillin–streptomycin (50 U/mL; Sigma) in 5% CO_2_/95% air at 37°C. Fresh culture medium was replaced on alternate days. To conduct the subsequent experiments, the cells were trypsinized at 60%–70% confluence. Mycoplasma was not detected in HK-2 cells.

A cell line of murine monocyte macrophage RAW 264.7 cells was purchased from the BCRC. In T75 flasks with 5% CO_2_/95% air at 37°C, cells were grown in DMEM supplemented with 10% fetal bovine serum containing 4 mM L-glutamine, 4500 mg/L glucose, 1 mM sodium pyruvate, 1500 mg/L sodium bicarbonate, and penicillin–streptomycin (50 U/mL; Sigma). Fresh culture medium was replaced on alternate days. The cells were trypsinized at 60%–70% confluence to conduct the experiments described below. Mycoplasma was not detected in RAW 264.7 cells. Cells were cultured from passages 3 to 10 in this study. Additionally, one or two replicates were performed in each passage.

### 2.3. Epithelial Cells from Primary Renal Tubules

A previously described method [24–26] was modified to assess mouse primary proximal tubule cells (mRTCs). Mice were sacrificed using cervical dislocation. An ice-cold Hank's balanced salt solution (Biological Industries, CT, USA) was placed in 15 mL conical tubes immediately after the kidneys were removed. Two razors were used to add the remaining cortical tissue into the dunce after removing the renal capsule, cortex, and excess fat. After breaking up the tissue with the plunger, the plunger was pushed five times into the bottom of the glass; then, the tissues were transferred to an ice-filled conical tube of 50 mL. A swinging bucket rotor was used to centrifuge the tubular tissues at 500 rpm and 4°C for 2 minutes. Once the supernatant is aspirated, the pelleted tissue is left intact while the supernatant is discarded.

A digestive medium containing 140 units/ml of collagenase I (Worthington, NJ, USA) in 20 mL HBSS and 15 mg of soybean trypsin inhibitor (Sigma, MO, USA) was added to the tube and incubated at 37°C for 15 minutes on an orbital shaker at 70 revolutions per minute. An incubator was used to mix the tubule suspension every five minutes with a 10 mL pipette. A 20 mL of cold horse serum was incubated with the tubule suspension after digestion to inactivate the enzymes and enrich the tubules. The tube was turned upside down until a uniform suspension was obtained. After settling for one minute, the tubules were discarded. A swinging bucket rotor was used to centrifuge the supernatant containing the proximal tubules for 2 minutes at 500 rpm. After washing with 10 mL of HBSS, the tubules were centrifuged for 2 minutes at 500 rpm. A 1-2 mL of culture medium was added with a sterile pipette to mix the pellet gently with the culture medium. A 40% Percoll/60% culture medium gradient was gently layered over the cortical tubule suspension. After centrifugation at 400 g for 10 minutes at 4°C, the tubules were collected. Two minutes of centrifugation at 500 rpm were performed on the tubules from the largest band in a 50 mL conical tube containing 20 mL of culture medium. As a final step, the tubule pellet was resuspended in 20 mL of MRPTC Culture Medium, which contained DMEM/F-12 culture media (Gibco, NY, USA) with insulin, transferrin, and selenium (5 grams/mL, respectively, Sigma), 0.05 mM hydrocortisone (Sigma), 50 mL L-ascorbic acid-2-phosphate (Wako, Tokyo, Japan), and 10% antibiotic/antimycotic solution (10,000 units/mL penicillin, 0.1 mg streptomycin, and 0.25 g/mL amphotericin B, Biological Industries, CT, USA).

**Figure 1:**
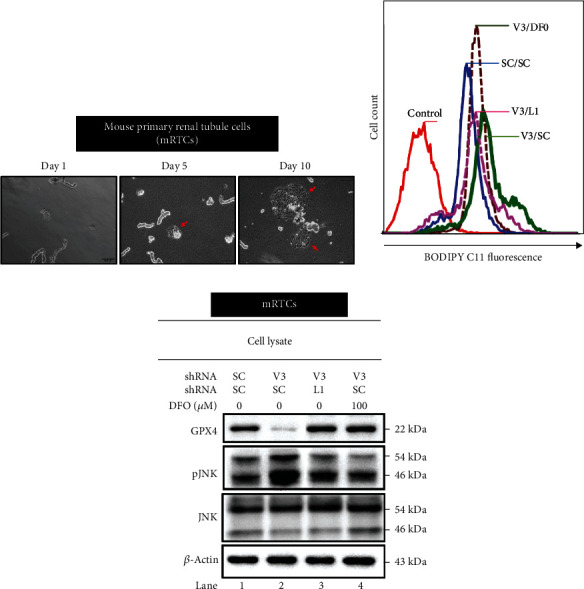
*vhl* mutant mediates the lipid ROS production, the expressions of GPX4 and JNK phosphorylation via LCN-2-dependent manner in mRTCs in a lipocalin 2 (LCN-2)-dependent manner. (a) The text describes the morphology and growth of mRTCs. The outgrowth of tubules is shown in the following examples. The arrow points to the tubule's tip, where the cells spread from day 5 to day 10. Scale bar: 100 *μ*M. (b) Changes in cellular lipid ROS levels of control (without C11-BODIPY staining) or V3/SC (mRTCs transfected with a vector expressing scrambled shRNA (SC) or shRNA specific for VHL, and shVHL3 (V3) in the absence of DFO, and V3/DFO (mRTCs were transfected with a vector expressing scrambled shRNA (SC) or shRNA specific for VHL, and shVHL3 (V3) in the presence of DFO) or V3/L1 (mRTCs transfected with a vector expressing specific for VHL and LCN-2). (c) Cell lysates were subject to western blot analysis with the indicated antibodies. *β*-Actin is used as a loading control.

Dilutional studies showed that diluted resuspended tubules were needed for optimal growth on different plates. Incubations were conducted at 37°C with 5% CO_2_ for 12-1.5 mL per well or 6-3 mL per well on Nunclon-treated tissue culture plates (Nalgene/Nunc International, Rochester, NY). A new MRPTC culture media was introduced daily to replace the old media. It usually takes between 5 and 12 days to achieve confluence (Figure 1(a)).

### 2.4. Transfection

According to the manufacturer's protocol and previous studies [4, 22], cells were transfected with shVHL3 (V3) or shLCN-2 (L1), kindly provided by Dr. T. Hsu (Graduate Institute of Biomedical Sciences, China Medical University, Taichung). In this study, we used the target sequences shVHL3 (V3), gagcctagtcaagcctgag, and shLCN-2 (L1), gactacaaccagttcgcc. A solution containing 4 *μ*g of plasmid was transfected with 5 × 10^5^ cells with a 100 V pulse for 10 mins and plated on six-well plates. Electroporated cells were cultured for 48 h and then analyzed for expression of VHL and LCN-2 by western blotting to determine transfection efficiency.

### 2.5. Western Blotting

SDS-PAGE was performed using 10% or 15% acrylamide gels with identical loading of proteins (30 *μ*g) per lane. We used polyvinylidene fluoride to transfer proteins from the gel after electrophoresis. During the transfer process, 350 mA was applied for 2 h. It was immersed in 5% nonfat milk for 1 h at room temperature while rotating at 75 rpm to block the membrane. The specific protein was then detected by incubating the membrane with primary antibodies overnight at 4°C. The following day, the membrane was washed three times in TBS buffer containing 0.2% Tween 20 (Sigma) and subsequently incubated for 1 h at room temperature with a 1 : 10000 dilution of the secondary antibody conjugated with horseradish peroxidase (HRP). After washing with TBS buffer containing 0.2% Tween 20, the western HRP substrate (Thermo Fisher Scientific; MA, USA) was linked to secondary antibodies. Finally, ChemiDoc XRS + System (Bio-Rad Laboratories; CA, USA) was used to visualize fluorescent protein signals.

### 2.6. Measurement of Lipid ROS

During incubation, cells were exposed to 2 mM C11-BODIPY 581/591 (Thermo Fisher Scientific) in a culture medium for 1 h before being washed with PBS. Flow cytometry was performed with cells collected after trypsinization (BD Biosciences; San Jose, CA, USA) at 488 nm and 517–527 nm wavelengths.

### 2.7. Measurement of Mitochondrial Iron

A red fluorescent indicator (RPA) was used to measure the mitochondrial iron concentration. First, viable cultured cells were incubated in Hank's balanced salt solution at 37°C for 15 min in the presence of a 10 *μ*M RPA. After incubation, the cells were washed twice with preheated Hanks' equilibration solution after removing the RPA reagent. The mitochondrial iron content was detected using a laser scanning microscope (Leica TCS SP5; Bensheim, Germany) with a red-fluorescence detection system.

### 2.8. Detection of IL-8 by Enzyme-Linked Immunosorbent Assay (ELISA)

After RAW cells were incubated with different conditioned medium, the collection of the supernatants was made for further investigation. Based on the experimental method provided by the reagent manufacturer, IL-8 levels in cell supernatants were measured by ELISA (#MBS7606860, IL-8 (Interleukin 8) Kit, MyBioSource, Inc., CA, USA). In this experiment, IL-8 concentration was calculated using standard curves provided with the kit, and the result was expressed as fold changes.

### 2.9. Statistical Analysis

Data were analyzed using a one-way or two-way analysis of variance (ANOVA using SPSS software, version 26.0, USA). *p* values < 0.05 were considered statistically significant (^∗^*p* < 0.05, ^∗∗^*p* < 0.01).

## 3. Results

### 3.1. *vhl* Mutant Induces Lipid ROS Production, Downregulates GPX4, and Upregulates LCN-2 in HK-2 Cells

**Figure 2:**
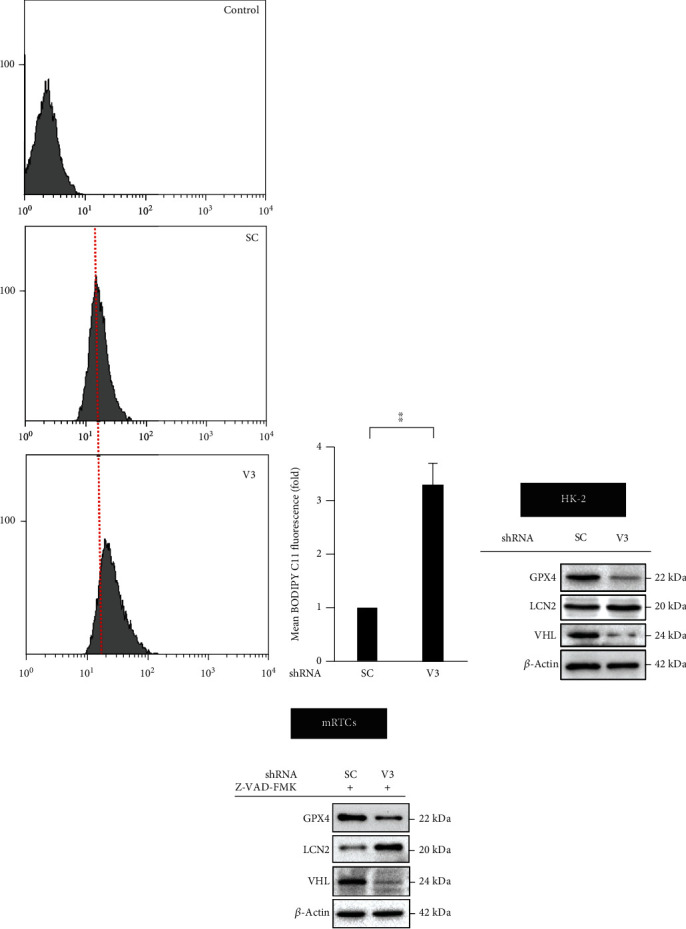
*vhl* mutant triggers ferroptosis and regulates the expression of glutathione peroxidase 4 (GPX4) and lipocalin 2 (LCN-2). (a) Changes in cellular lipid reactive oxygen species (ROS) level of control (without C11-BODIPY staining), as well as HK-2 cells, transfected with a vector expressing scrambled shRNA (SC) or shRNA specific for VHL: shVHL3 (V3) as described in the Methods section. (b) The ROS levels are presented as mean fluorescence intensity. Error bars represent the standard deviation from three independent replicates (*n* = 3). ^∗∗^*p* < 0.01. Cell lysates isolated from HK-2 (c) and mRTCs (d) were subject to western blot analysis with the indicated antibodies. *β*-Actin is used as a loading control. mRTCs were treated with 20 *μ*M Z-VAD-FMK to rule out the apoptotic effect.

Results indicated that *vhl* mutation caused excess lipid ROS production (Figures 2(a) and 2(b)). Our previous study demonstrated that VHL regulates chronic inflammation in ccRCC progressionvia the LCN-2–ROS pathway [22]; however, the critical role of *vhl* mutations in LCN-2 regulation of ccRCC formation via the ferroptotic-inflammatory pathway is unclear. We proposed that *vhl* mutation caused ferroptosis and inflammation via LCN-2 overexpression in HK-2 cells. As shown in Figure 2(c), our results revealed that a decrease in GPX4 and an increase in LCN-2 were detected in the VHL-knockdown (V3) HK-2 cells (Figure 2(c)) and mouse primary proximal tubule cells (mRTCs) (Figure 2(d)).

**Figure 3:**
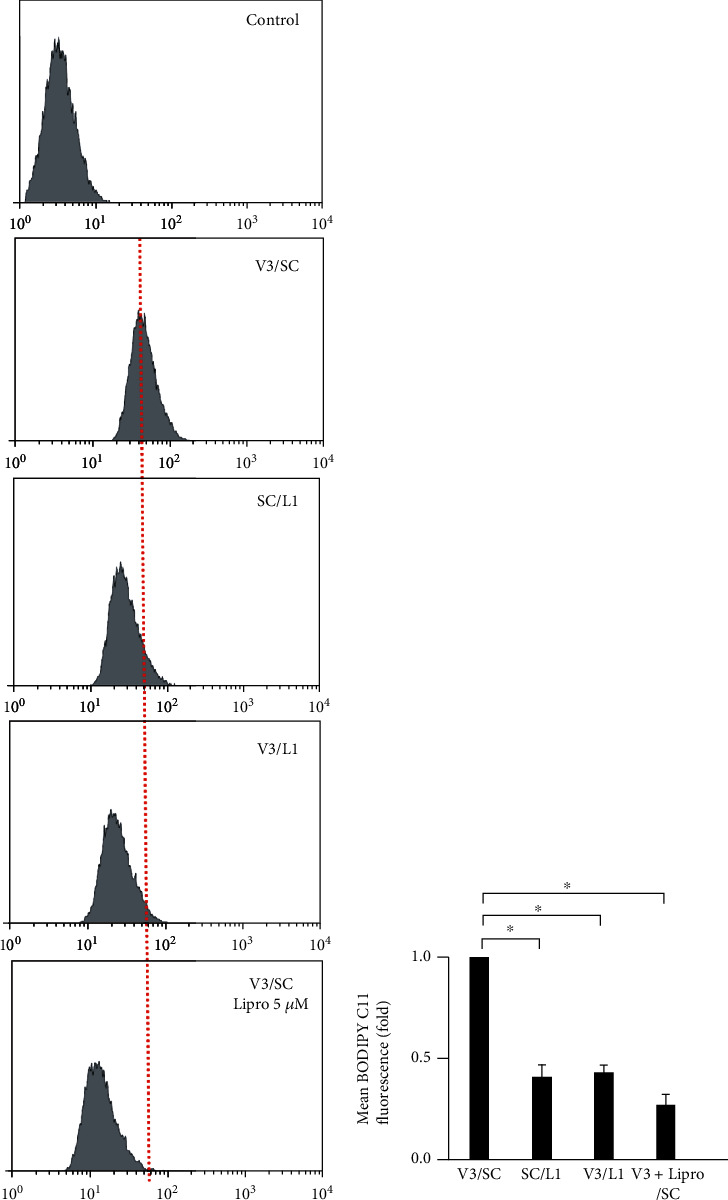
The *vhl* mutant increased lipid reactive oxygen species (ROS) production in a lipocalin 2 (LCN-2)-dependent manner. (a) Changes in cellular lipid ROS levels of control (without C11-BODIPY staining), as well as HK-2 cells, transfected with a vector expressing scrambled shRNA (SC) or shRNA specific for VHL: shVHL3 (V3) or shLCN-2 (L1), as described in the Methods section. V3/SC Lipro-1 (5 *μ*M) indicated HK-2 cells were transfected with shVHL3 and scrambled shRNA (SC) in the presence of 5 *μ*M liproxstatin-1. (b) Lipid ROS levels are presented as the mean fluorescence intensity. Error bars represent the standard deviation from three independent replicates (*n* = 3). ^∗^*p* < 0.05.

To evaluate the role of LCN-2 in regulating lipid ROS in *vhl* knockdown HK-2 cells, we detected the level of lipid ROS in the absence or presence of LCN-2 in our cell model. Results demonstrated a decrease in the level of lipid ROS in the presence of VHL and the absence of LCN-2 (SC/L1). Similar results were observed in the absence of LCN-2 in *vhl* knockdown HK-2 cells (V3/L1) (Figures 3(a) and 3(b)). The Lipro-1, a small-molecule inhibitor for ferroptotic activity, was used to study the level of lipid ROS to further confirm whether the *vhl* mutant caused lipid ROS accumulation via ferroptosis. Results showed that Lipro-1 (5 *μ*M) alleviated lipid ROS overproduction in *vhl*-mutant HK-2 cells (V3 + Lipro-1/SC) (Figures 3(a) and 3(b)).

### 3.2. *vhl* Mutant Regulates GPX4 Expression and JNK Phosphorylation via LCN-Dependent Ferroptosis

**Figure 4:**
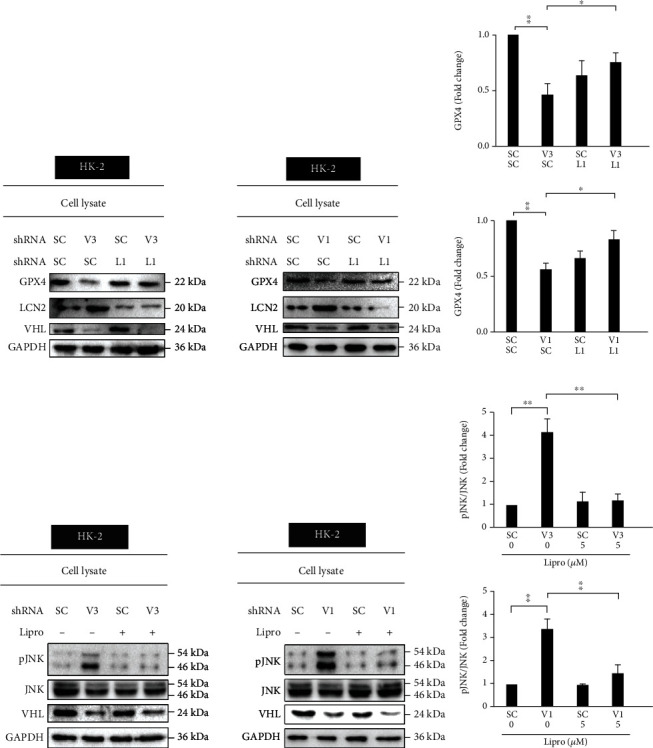
*vhl* mutant regulated the expression of glutathione peroxidase 4 (GPX4) and induced an inflammatory response via ferroptosis-dependent pathways. (a) HK-2 cells were transfected with a vector expressing scrambled shRNA (SC) or shRNA specific for VHL: shVHL3 (V3, left panel; V1, right panel) or shLCN-2 (L1), as described in the Methods section. Cell lysates were subject to western blot analysis with the indicated antibodies. Glyceraldehyde 3-phosphate dehydrogenase (GAPDH) was used as a loading control. The expression states of LCN-2 and VHL were measured by the levels of the LCN-2 and VHL proteins, respectively. The *vhl* knockdown caused decreased GPX4 levels, reversed in shVHL1 (V3, left panel; V1, right panel) and shLCN-2 (L1) knockdown cells. (b) The quantitative results for specific proteins as determined by ImageJ. The data were presented as the mean ± SD of the results for 3 independent experiments.^∗^*p* < 0.05, ^∗∗^*p* < 0.01. (c) The *vhl* knockdown (V3, left panel; V1, right panel) caused increased p-JNK levels in the absence of liproxstatin-1 treatment, which was reversed in the presence of liproxstatin-1 treatment (5 *μ*M). (d) Triplicates of the experiments were quantified using ImageJ. All data are presented as mean ± SD. ^∗^*p* < 0.05, ^∗∗^*p* < 0.01.

This study showed that LCN-2 knockdown significantly attenuated a decrease in GPX4 expression in *vhl*-mutant HK-2 cells by western blotting (Figures 4(a) and 4(b)). Moreover, under c-Jun NH_2_-terminal kinase activation, phosphorylated JNK (p-JNK) plays a key role in mediating renal tubulointerstitial inflammation [27]. To further confirm whether *vhl* mutant affects JNK phosphorylation via ferroptosis, the small-molecule ferroptotic inhibitor Lipro-1 was used to study the level of p-JNK. Results indicated that Lipro-1 (5 *μ*M) alleviated JNK phosphorylation in *vhl*-mutant HK-2 cells (Figures 4(c) and 4(d)). These results directly showed that inhibition of ferroptotic function prevented ferroptosis–GPX4-mediated inflammation.

### 3.3. DFO Alleviates *vhl* Mutant-Enhanced Production of Mitochondrial Iron and Lipid ROS and JNK Phosphorylation

**Figure 5:**
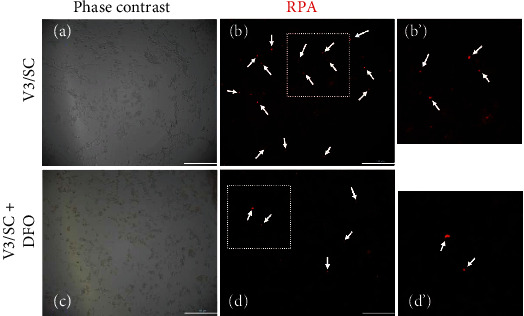
Deferoxamine mesylate (DFO) reverses the increase in mitochondrial iron accumulation in *vhl*-mutant HK-2 cells. The amount of mitochondrial labile iron ion is measured by staining the mitochondria with red fluorescent indicator (RPA) and the iron chelator DFO. (a) HK-2 cells were transfected with a vector expressing scrambled shRNA (SC) or shRNA specific for VHL, shVHL3 (V3), as described in the Methods section. The phase-contrast image is detected in the absence of DFO in *vhl* knockdown (V3) HK-2 cells. (b) Arrows indicate the positive signal of RPA fluorescence in the absence of DFO in *vhl* knockdown (V3) HK-2 cells. (c) The phase-contrast image is detected in the presence of DFO in *vhl* knockdown (V3) HK-2 cells. (d) Arrows indicate the positive RPA fluorescence signal in the presence of DFO in *vhl* knockdown (V3) HK-2 cells. (b') and (d') were enlarged from (b) and (d), respectively. The bar indicates 100 *μ*M.

**Figure 6:**
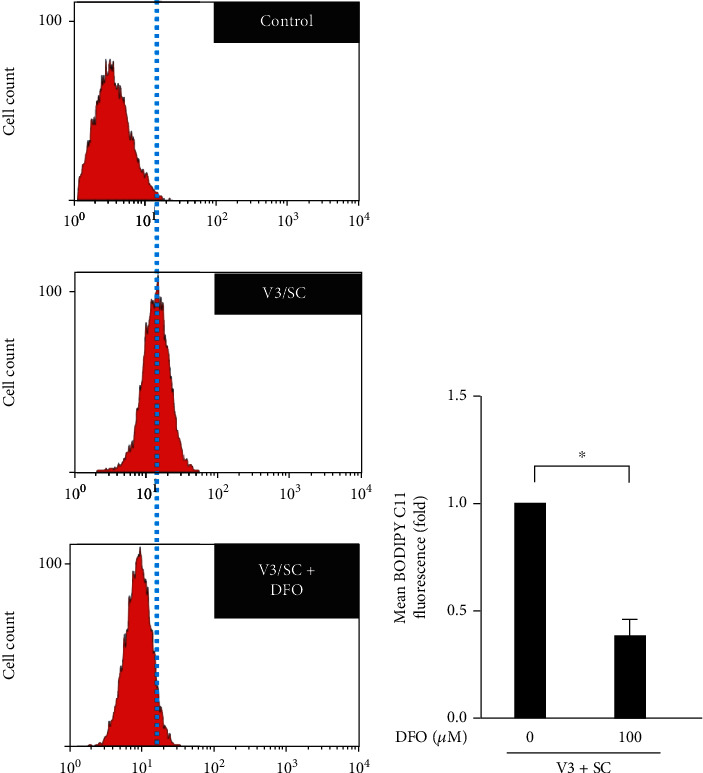
Deferoxamine mesylate (DFO) reverses lipid reactive oxygen species (ROS) accumulation in *vhl*-mutant HK-2 cells. (a) Changes in cellular lipid ROS levels of control (without C11-BODIPY staining) or V3/SC (HK-2 cells transfected with a vector expressing scrambled shRNA (SC) or shRNA specific for VHL, and shVHL3 (V3) in the absence of DFO, and V3/SC + DFO. HK-2 cells were transfected with a vector expressing scrambled shRNA (SC) or shRNA specific for VHL, and shVHL3 (V3) in the presence of DFO. (b) Lipid ROS generation is presented as the mean fluorescence intensity. Error bars represent the standard deviation from three independent replicates (*n* = 3). ^∗^*p* < 0.05.

**Figure 7:**
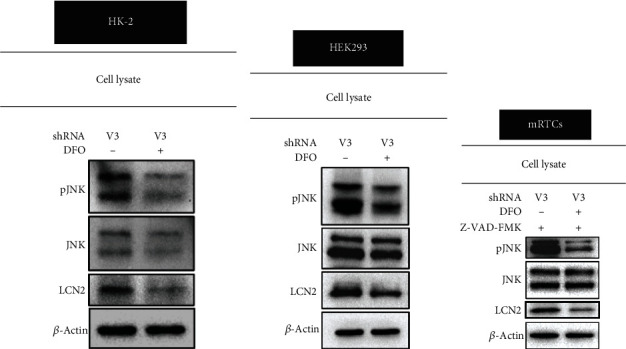
Deferoxamine mesylate (DFO) reverses *vhl* mutant upregulated p-JNK and lipocalin 2 (LCN-2). (a) HK-2, (b) HEK293, and (c) mRTCs cells were transfected with a vector expressing shRNA specific for VHL: shVHL3 (V3) with and without DFO treatment, as described in the Methods section. Cell lysates were subject to western blot analysis with the indicated antibodies. *β*-Actin is used as a loading control. mRTCs were treated with 20 *μ*M Z-VAD-FMK to rule out the apoptotic effect.

The possible correlation between cellular iron content and *vhl* mutation in HK-2 cells was examined. DFO attenuated the effects of the *vhl* mutation (V3/SC) and caused the overproduction of mitochondrial iron in HK-2 cells, as measured by RPA staining (Figures 5(a)–5(d)). The study further shows that the DFO reversed the increase in the production of lipid ROS in *vhl*-mutant HK-2 cells (Figures 6(a) and 6(b)). The increase in JNK phosphorylation in *vhl*-mutant HK-2 cells (Figure 7(a)), HEK-293 cells (Figure 7(b)), and mRTCs (Figure 7(c)) were reversed after DFO treatment, suggesting that *vhl* mutant-induced production of mitochondrial iron and lipid ROS, as well as inflammation, was associated with ferroptosis in an iron-dependent manner.

### 3.4. *vhl* Mutant Sensitizes RAW Cell Polarization via LCN-2-Dependent Manner

**Figure 8:**
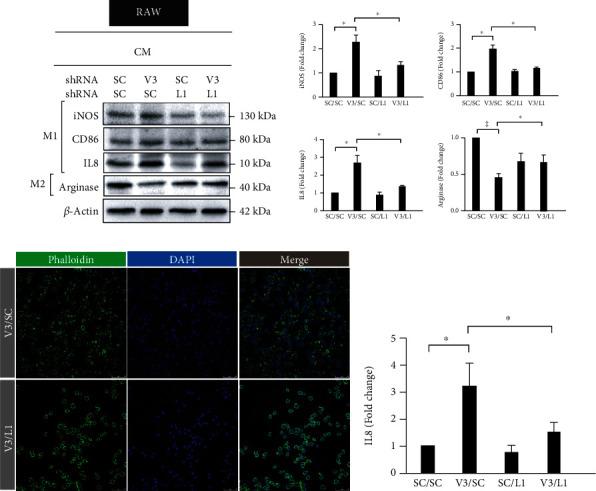
*vhl* mutant sensitizes macrophage polarization in a lipocalin 2 (LCN-2)-dependent manner. (a) The polarization of macrophages is mediated by LCN-2 in *vhl*-mutant HK-2 cells. The HK-2 cells or without the knockdown of VHL (V3) or without (SC), and with knockdown of LCN-2 (L1) or without (SC), are assayed for their ability to recruit monocyte/macrophage RAW 264.7 cells. The M1- and M2-specific markers are detected using the indicated antibodies. *β*-Actin is used as a loading control. An image of the right panel shows the quantitative results for specific proteins as determined by ImageJ. The data were presented as the mean ± SD of the results for 3 independent experiments. ^∗^p < 0.05, ^∗∗^p < 0.01. (b) A pancake-like cell shape is associated with macrophage polarization toward an M2 phenotype in those treated with V3/L1-conditioned medium (cm). Fluorescence images of phalloidin staining in green and DAPI nuclear counterstaining in blue. Scale bar: 50 *μ*M. CM: conditioned medium. (c) The level of IL-8 was detected by ELISA. Error bars represent the standard deviation from three independent replicates (*n* = 3). ^∗^*p* < 0.05.

The *vhl* mutant effects on RAW cell polarization was examined, we detected the expressions of inducible nitric oxide synthase (iNOS), CD86, and interleukin- (IL-) 8, the typical M1-like macrophage markers, as well as arginase, a typical M2-like macrophage marker, respectively. As shown in Figure 8, *vhl* mutant resulted in a marked increase in the expressions of iNOS, CD86, and IL-8 but a significantly decrease in arginase expression (Figure 8(a)). The LCN-2 knockdown reversed the increase in the expressions of iNOS, CD86, and IL-8 in *vhl*-mutant HK-2 cells (V3/L1) (Figure 8(a)). Moreover, the LCN-2 knockdown reversed the decrease in the expression of arginase in *vhl-* and *LCN2*-mutant HK-2 cells (V3/L1) (Figure 8(a)). On the other hand, actin depolymerization was not observed in RAW cell that cultured in conditional medium cultured in HK-2 cells with VHL mutations (V3/L1) (Figure 8(b), upper panel). However, a pancake-like shape was observed in RAW cell that cultured in *vhl-* and *LCN2*-mutant HK-2 cells (V3/L1) (Figure 8(b), lower panel). To further confirm the release of IL-8 in RAW cell that cultured in the indicated conditioned medium, the level of IL-8 was measured by ELISA. Results showed that *vhl* mutant caused the increasing in the release of IL-8, but was reversed in LCN-2 knockdown condition (Figure 8(c)). Thus, we suggested that *vhl* mutant stimulated polarization toward an M1-like phenotype in an LCN-2-dependent manner.

### 3.5. *vhl* Mutant Regulates Lipid ROS Accumulation and Expressions of GPX4 and JNK Phosphorylation via LCN-2-Dependent Manner in mRTCs

The levels of lipid ROS and the expressions of GPX4 and p-JNK in *vhl*-mutant mRTCs were determined to further verify our hypothesis in this study. Results indicated that the increase in lipid ROS production was detected in *vhl*-mutant mRTCs (Figure 1(b), V3/SC, green curve) but was reversed in *vhl* and *LCN2*-mutant mRTCs (Figure 1(b), V3/L1, purple curve), and was also detected in the presence of DFO in *vhl*-mutant mRTCs (Figure 1(b), V3/DFO, brown curve). On the other hand, results also showed that LCN-2 knockdown (Figure 1(c), V3/L1, lane 3) and DFO (Figure 1(c), lane 4) significantly attenuated a decrease in GPX4 expression in *vhl*-mutant mRTCs by western blotting. The increase in JNK phosphorylation was observed in *vhl*-mutant mRTCs (Figure 1(c), lane 2) but was reversed after LCN-2 knockdown (Figure 1(c), lane 3) and DFO treatment (Figure 1(c), lane 4). Taken together, we suggested that *vhl* mutant-induced production of lipid ROS and inflammation was associated with ferroptosis in an iron-dependent manner.

### 3.6. *vhl* Mutant Results in ACSL4, HMGB1, and Lon Upregulation in HK-2 and mRTCs

**Figure 9:**
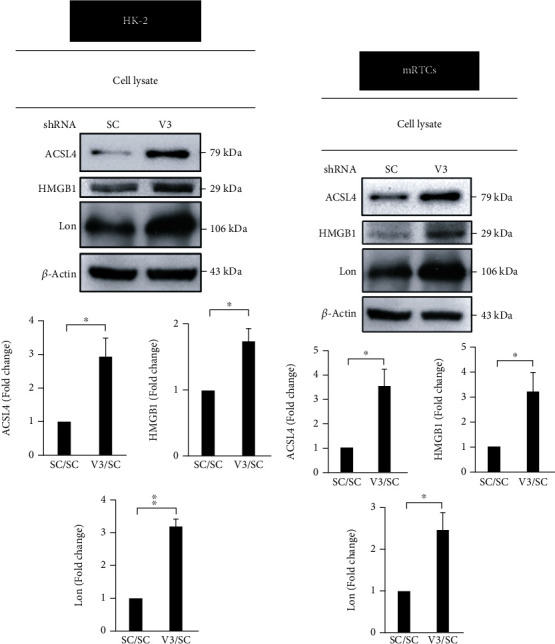
*vhl* mutant mediates the expressions of ACSL4, HMGB1, and Lon. Changes in the expressions of ACSL4, HMGB1, and Lon in *vhl*-mutant HK-2 (a) and mRTCs (b). Cell lysates were subject to western blot analysis with the indicated antibodies. *β*-Actin is used as a loading control. *β*-Actin is used as a loading control. An image of the lower panel shows the quantitative results for specific proteins as determined by ImageJ. The data were presented as the mean ± SD of the results for 3 independent experiments. ^∗^*p* < 0.05, ^∗∗^*p* < 0.01.

Acyl-CoA synthetase long-chain family member 4 (ACSL4) and high mobility group box 1 (HMGM1) have been reported that play a critical role in regulation of ferroptosis [28], however, the effect of *vhl* mutant on the expressions of ACSL4 and HMG1 in renal tubular cells are still unclear. As shown in Figure 9, the increasing in the expressions of ACSL4 and HMG1were observed in *vhl*-mutant HK-2 and mRTCs. Moreover, we previously demonstrated that hypoxia-induced ROS-dependent apoptosis in cardiomyocytes is regulated by mitochondrial Lon protease (Lon) [29]. Nonetheless, comprehensive and detailed studies are needed to examine the role of Lon in ferroptosis in *vhl*-mutant HK-2 and mRTCs. Results indicated that Lon was upregulated in in *vhl*-mutant HK-2 and mRTCs (Figure 9).

## 4. Discussion

A toxic iron condition, lipid peroxidation, and damage to the plasma membrane are all responsible for ferroptosis, regulated necrosis, or cell death. Moreover, ferroptotic cell death is implicated in several physiological and pathological processes accompanied by dysregulation of the immune system, including inflammation [30]. The functional version of the von Hippel-Lindau gene (*vhl*) blocks ferroptosis by reversing cell's metabolism to an oxidative one and stimulating fatty acid breakdown [31]. Maxwell et al. reported that hypoxia-inducible factor- (HIF-) *α* subunits are stabilized and activated in VHL-defective cells. Oxygen-dependent instability was restored through VHL reexpression. In hypoxic HIF-1 DNA-binding complexes, VHL coimmunoprecipitates with HIF-*α* subunits. The dissociation of HIF-1 from VHL is detected in cells after treatment with iron chelators (DFO) or cobaltous chloride. According to these results, iron is an important determinant in the interaction between HIF-1 and VHL and is required for the oxygen-dependent degradation of HIF-*α* subunits [32]. However, some conflicting results suggest that loss of VHL induces transcriptional activation, resulting in increased uptake of iron-bound transferrin by HIF-1 in renal carcinoma cells. An increase in iron availability does not compromise the ability of VHL-defective cells to resist oxidative stress or enhance cell proliferation [33]. In a previous study, total iron and ferritin levels in the liver of VHL knockout mice were significantly lower than in wild-type mice. Moreover, the VHL–HIF pathway is important for regulating hepcidin and iron homeostasis. The VHL–HIF pathway mobilizes iron through coordinated downregulation of hepcidin and upregulation of erythropoietin and ferroportin [34].

In this study, we attempted to determine the role of VHL in ccRCC. LCN-2 is a well-established biomarker for acute and chronic kidney diseases and is rapidly upregulated after renal tubular injury [35, 36]. LCN-2 expression clustered in patients with high and low levels of ccRCC and was associated with poor patient survival. An overexpression of LCN-2 protein was observed in tumors compared with adjacent healthy tissue, and iron was found to accumulate in LCN-2. In vitro, iron-loaded LCN-2 inhibited tumor growth, whereas iron-free LCN-2 caused adverse effects [37]. Furthermore, Chaudhary et al. reported that a monoclonal antibody inhibits LCN-2 function in vitro and in xenograft models to reduce tumor progression and chemoresistance via ferroptosis in colon cancer [38]. A previous study showed that iron-loaded LCN-2 stimulates the ROS–Nfr2 axis and promotes SLC7A11 expression in response to integrated stress responses during erastin-induced ferroptosis [39]. These findings reveal the mechanistic details of the initiation of iron-loaded LCN-2-mediated tumor prosurvival pathways. They may facilitate the development of novel therapeutic strategies to reduce tumor growth by blocking the iron-binding ability of LCN-2.

Our studies reveal the role of LCN-2 in regulating the accumulation of mitochondrial iron and intracellular lipid ROS in response to ferroptosis and polarization of RAW cells in *vhl*-mutant HK-2 cells. However, Liu et al. proposed that LCN-2 might protect against acute pancreatitis by inhibiting ferroptotic processes in conditional LCN-2 knockout mice (LCN-2^Pan−/−^) [40]. Some studies have revealed a high level of LCN-2 expression in patients with inflammatory breast cancer (IBC) and that it is essential for tumor growth and skin invasion in mice with IBC [41]. Similar results showed that small interfering RNA and small-molecule inhibitors target LCN-2 in inflammatory breast cancer cells [42]. It has also been noted that pancreatic cancer cachexia is associated with increased circulating levels of LCN-2, an anorexic molecule that contributes to muscle and fat wasting [43]. Kim et al. demonstrated that IL-6 was significantly associated with the upregulation of LCN-2 mRNA and protein levels in murine colitis-associated cancer models and human colorectal cancer cells. Moreover, the authors discovered that LCN-2 upregulation by IL-6 is reduced when NF-*κ*B and STAT3 (signal transducer and activator of transcription 3) are inhibited with specific inhibitors and small interfering RNA. According to report assay results, IL-6 induces LCN-2 gene promoter activity by activating STAT3 and NF-*κ*B. LCN-2 is induced by IL-6 and activates the NF-*κ*B/STAT3 pathway, which enhances cell survival [44]. According to these data, LCN-2 could be a potential therapeutic target to improve appetite during carcinogenesis.

The most abundant inflammatory cells that infiltrate tumors are macrophages, which play a key role in regulating chronic inflammation [45, 46]. Macrophages are one of the most abundant immune cells in the tumor microenvironment of solid tumors. Their presence is associated with a reduced chance of survival in most cancers [47]. Tumor-infiltrating macrophages can acquire phenotypic and functional properties distinct from those within healthy tissues, reflecting their extreme plasticity in the diminution of antitumor activity and induction of tumor-supporting functions, these are termed tumor-associated macrophages with M2-like characteristics [46, 48]. LCN-2 plays a role as an anti-inflammatory factor in regulating M1/M2 polarization via modulating NF-*κ*B–STAT3 loop activation in the isolated LCN-2-deficient bone marrow-derived macrophages [49]. However, conflicting results demonstrated that LCN-2 promotes polarization of M1 macrophages in a mouse model with cardiac ischemia-reperfusion injury [50]. Jung et al. concluded that LCN-2 expression in tissue-infiltrating macrophages is critical for kidney-intrinsic cytoprotection during ischemia reperfusion injury [51]. A mouse model of pneumococcal pneumonia transfected with LCN-2 revealed a reduced inflammatory response and impaired bacterial clearance. IL-10 production by macrophages is induced by LCN-2, skewing macrophage polarization in a STAT3-dependent manner [52]. Increased levels of inflammatory monocytes with no effect on neutrophil recruitment have been linked to a decrease in plaque size in LCN-2-deficient animals during the early stages of lesion development [53]. However, our data indicated that the *vhl* mutant stimulated polarization toward an M1-like phenotype in an LCN-2-dependent manner. Therefore, the different results of LCN-2 in regulating macrophage polarization may be due to differences in types of cells, experimental systems, or even immortalized or primary cells.

In this study, we used HK-2 and mRTCs as cell models verifying the effect of the VHL mutant on the regulation of chronic inflammation within the LCN-2–ferroptosis pathway. The chronic inflammatory effect of *vhl* mutation via LCN-2–ferroptosis pathway needs to be further confirmed with experimental animals or clinical specimens in the future.

## 5. Conclusions

**Figure 10:**
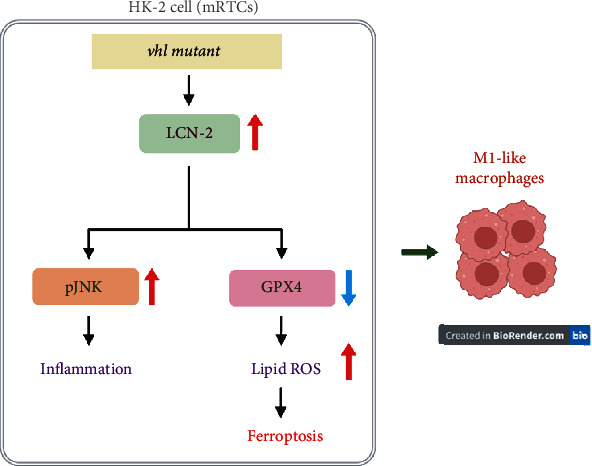
Schematic of the mechanisms. Knockdown of the *vhl* gene (*vhl* mutant) in HK-2 cells induces lipocalin 2 (LCN-2) overexpression, p-JNK upregulation, lipid reactive oxygen species (ROS) accumulation, and glutathione peroxidase 4 (GPX4) downregulation, leading to inflammation and ferroptosis. Moreover, the *vhl* mutant regulated the polarization of macrophages. These results reveal the regulatory effect of VHL on inflammation in HK-2 cells via the LCN-2–ferroptosis pathway.

Our results indicated that VHL mutation caused overproduction of lipid ROS, pJNK upregulation, and GPX4 downregulation, as well as M1-like polarization, but an LCN-2 knockdown could reverse this process. Overall, our study revealed that VHL has an important regulatory role in ccRCC progression by regulating chronic inflammation via the LCN-2-ferroptosis pathway (Figure 10). Our findings offer novel insights into potential therapeutic targets and strategies for preventing ccRCC.

## Data Availability

The original data used to support the findings of this study are included in the article.
